# Optimizing growth conditions in vertical farming: enhancing lettuce and basil cultivation through the application of the Taguchi method

**DOI:** 10.1038/s41598-023-33855-z

**Published:** 2023-04-25

**Authors:** Hadis Farhangi, Vahid Mozafari, Hamid Reza Roosta, Hossein Shirani, Mosen Farhangi

**Affiliations:** 1 Department of Soil Science and Engineering, Vali-e-Asr University of Rafsanjan, Rafsanjan, Iran; 2 Department of Horticulture Sciences, Arak University, Arak, Iran; 3 Department of Thematic Studies - Technology and Social Change, Linköping University, Linköping, Sweden

**Keywords:** Plant sciences, Plant development, Leaf development

## Abstract

This paper reports on the findings of an experimental study that investigated the impact of various environmental factors on the growth of lettuce and basil plants in vertical farms. The study employed the Taguchi method, a statistical design of experiments approach, to efficiently identify the optimal growth conditions for these crops in a hyper-controlled environment. By reducing the time and cost of designing and running experiments, this method allowed for the simultaneous investigation of multiple environmental factors that affect plant growth. A total of 27 treatments were selected using the Taguchi approach, and the signal to noise ratio was calculated to predict the optimal levels of each environmental condition for maximizing basil and lettuce growth parameters. The results showed that most of the parameters, except for EC and relative humidity for certain growth parameters, were interrelated with each other. To validate the results, confirmation tests were conducted based on the predicted optimal parameters. The low error ratio between expected and predicted values (1–3%) confirmed the effectiveness of the Taguchi approach for determining the optimal environmental conditions for plant growth in vertical farms.

## Introduction

The global demand for sustainable, high-quality food production has led to a growing interest in vertical farming, an innovative agricultural approach that utilizes controlled environments to maximize crop yields^1^. An increased interest from governments, investors and practitioners in vertical farming is accelerating the expansion of the vertical farming sector^2^. Vertical farms have the potential to contribute to sustainability and resilience of food systems by achieving a higher yield than traditional agriculture and using less water and land resources^1^. Many vertical farms use hydroponic cultivation methods and technologies for plant’s nutrient management and controlling the growth environment of plants^3^. Optimizing the nutrient management in the hydroponic system and environmental conditions is necessary to improve the quality and quantity of the produced crops^4^. To use the potentials of the technologies that are used in vertical farming for improving the quality and quantity of the crops, gathering enough data over the needs of cultivated plants, and optimizing the factors influences the cultivation process is essential^5,6^. Previous research has studied the factors that influence the production of different types of leafy vegetables in hydroponic systems and vertical farms^7–10^. However, most of these studies merely focused on one factor or combination of two or three factors at the same time^11–13^. Investigating multiple influential factors on growth of plants is a challenging task^14^. It requires an experiment with numerous combinations of factors or conducting many experiments with one factor at a time. Due to complexity of such experiments these experiments are hardly practical. Additionally, such studies are very time consuming and costly^15^.

In an effort to optimize the growth of lettuce and basil plants in vertical farms, the present study utilizes the Taguchi method, a highly efficient experimental design approach, to identify the ideal environmental conditions for these crops. Through this robust statistical method, we tend to simultaneously examine multiple environmental factors that affect plant growth, such as light intensity, temperature, carbon dioxide levels, and nutrient solutions, and determine their optimal levels for promoting the growth of lettuce and basil in vertical farming systems. This research aims to contribute to the field of vertical farming, ultimately improving crop production and sustainability in this rapidly expanding industry.

This paper investigates the possibilities of applying Taguchi method to reduce the number of experiments and analyse the effect of several factors at same time^16,17^. Design of experiments (DOE) using Taguchi method is a combination of statistical and engineering methods and are a Factorial based approach. In this approach, Taguchi orthogonal array (OA) analyse variable factors in different levels by designing a small number of experiments using the linear graphs^18,19^. The obtained results can be expanded for general conclusion to find the nearly optimized level of each factor using a simple process^20,21^. In this study, six important factors affected the production of lettuce and basil in vertical farms as the representative of cold and warm climate plants were selected. After conducting an extensive review of the literature concerning the key factors influencing growth parameters in Controlled-Environment Agriculture (CEA), six crucial factors have been identified (Table 1): electrical conductivity (EC) of the nutrient solution, day and night temperatures, relative humidity, CO_2_ concentration, and LED light recipes. The significance of these six factors is thoroughly examined and discussed based on previous studies in the subsequent sections.

**Table 1:** Influential factors on growth of plants in vertical farms.

Factor	Description	Reference
Light	Effect of LED light ratio on lettuce parameters	Izzo, Mickens^22^
	Effect of LED and light intensity on lettuce	Legendre and van Iersel^23^
	Effect of LED on leafy green	Wong, Teo^25^
	Effect of LED light ratio on lettuce	Meng and Runkle^26^
	Effect of light intensity on lettuce and basil	Pennisi, Pistillo^38^
Nutrition	Effect of EC on lettuce	Kappel, Boros^30^
	Effect of EC on basil	Solis-Toapanta, Fisher^31^
	Effect of temperature on lettuce	Gent^39^
Temperature	Temperature optimising in indoor gardening	Perone, Orsino^32^
	Effect of temperature on basil	Walters and Currey^40^
	Effect of night temperature on lettuce	Jeong, Kim^34^
CO_2_	Effect of CO_2_ on leafy greens	Singh and Bruce^9^
	Effect of CO_2_ on vegatable	Dong, Gruda^35^
	Effect of CO_2_ on lettuce	Becker and Kläring^36^
Relative humidity	The effect of RH on lettuce	Vanhassel, Bleyaert^37^
CO_2_ and temperature	Interaction between two factors on basil (less impact of CO_2_)	Barickman, Olorunwa^41^
Light and temperature	Interaction between light intensity and temperature on lettuce	Carotti, Graamans^42^
Temperature and nutrition	Interaction between temperature and K (less impact of K) on lettuce	Sublett, Barickman^5^
EC and light	Interaction between light and EC on basil	Walters and Currey^33^

LED light recipes is one of the factors chosen for this experimental study. LED lighting, due to its energy-efficiency and generating less heat than other lighting sources, promoting a cooler environment and reducing the risk of heat stress for the plants is one the most used light sources used in vertical farming sector^22,23^. LED lighting also impacts plant morphology, yield, and quality, allowing control over the growth cycle of plants, such as promoting vegetative growth or inducing flowering^24^. LED lighting is also an important factor in hydroponic culture, offering precise control over the spectrum of light to optimize plant growth and development^22,23,25,26^. Although LEDs have been confirmed to be advantageous for plant cultivation, but to optimize their effectiveness, it’s important to study the impact of different light spectra and intensities for each type of plant. Some studies have focused on the influence of light spectrums and wavelengths, with recent results showing that white LED can be beneficial for plant growth, particularly when used with supplementary red and far red lights^27,28^. Determining the optimal light intensity or photon flux density (PPFM) is also important in finding a suitable lighting recipe for plants^29^.

EC is another critical factor in hydroponic vertical farming which was studied in this experiment. It reflects the concentration of dissolved salts in the nutrient solution, directly affecting the availability of nutrients to the plants. Optimal EC levels vary depending on the specific cultivar of lettuce and basil^8^. High EC levels can cause salt accumulation and root damage, while low EC levels can result in nutrient deficiencies and poor plant growth^30,31^. The optimal EC level for plant growth and development depends on the cultivation system and plant type. In ebb-flow hydroponic systems, where the roots of the plant float in the nutrient solution, plants require less EC than other systems^8^. A recent study by Hosseini et al.^8^ found that the optimal EC level for basil and lettuce growth in ebb-and-flow systems was 1.2 and 0.9 dS m^−1^, respectively.

Day and night temperature are also important factors that were included in this study, as temperature affects plant growth, development, and metabolism. The best temperature for the growth of basil and lettuce varies depending on the stage of growth and the specific cultivar being grown. In general, cooler temperatures are preferred, but these plants can tolerate some level of heat stress. It is important to note that different cultivars of basil and lettuce may have differing temperature requirements, so it's essential to research the ideal conditions for the specific cultivar that are growing^32,33^. Optimal temperature during the day and night for lettuce and basil minimize temperature fluctuations, promote photosynthesis and plant growth during the day, and reduce respiration rates and plant stress at night. Extreme temperatures can negatively impact plant growth and development, causing heat or water stress, reduced nutrient uptake, or even damage or death to the plants^33,34^.

CO_2_ enrichment is essential for hydroponic cultivation of lettuce and basil, as it is a key component of photosynthesis, therefore it was considered for experimentations in this study. Maintaining an optimised CO_2_ concentration promotes efficient light energy conversion into carbohydrates and improves plant photosynthesis, respiration, and nutrient uptake, ultimately impacting leaf size and flavour^35,36^.

Relative humidity affects plant growth, transpiration rates, and nutrient uptake in hydroponic systems used in vertical farms and therefore it is an important factor that affects the growth conditions of the plants^37^. Proper humidity levels, reduce water loss through transpiration and improve nutrient uptake by the plants while limiting disease development and pest pressure^37^.

Therefore, the appropriate control g and adjustment EC, LED lighting, CO_2_ concentration, relative humidity, and day and night temperature are essential factors for optimizing plant growth and yield in vertical farming. For the experimental study Minitab was used to design the orthogonal table and the data were analysed by using Taguchi method. The main aim of the paper was top explore the best conditions for cultivation of lettuce in controlled-environment vertical farms.

## Materials and methods

### Vertical farms and plant growth chambers

The study was done at Vegger vertical farming facility in World Food Centre in Ede, The Netherlands. The experiments were performed by using Vegger’s modular vertical farming units that were installed in controlled environment grow chambers in World Food Centre. The used systems had an ebb-flow hydroponic cultivation system.

The vertical farming units are utilized for both commercial and research purposes for cultivation of vegetables and herbs. The experiments were conducted using 5-level vertical farms, with a distance of 50 cm between each level and 12 cm between each plantation pot. The roots were irrigated five times a day, with a duration of 10 min each time.

Each level had space for 32 plantation pots (size of each pot: 9 cm H × 9 cm W × 9 cm L). The used cultivation substrates were rockwool blocks for lettuce and vermiculite for basil.

The grow chambers were small dark rooms equipped with vertical farms and equipment for controlling and regulating the environmental conditions. The light source of the units was provided by LED top lighting. The percentage of each radiation and PPFD (Photosynthetic Photon Flux Density) were adjusted with a light control system. The EC and pH levels of nutrient solutions were adjusted by a dosing machine which is an automated system designed to control and adjust pH and EC of the solution. Additionally, a controlled environment monitoring, and adjustment system was used in the grow chambers to regulates environmental conditions by creating equal growing conditions for all experiments. This system adjusted factors such as EC and pH levels, CO2 levels, humidity, temperature, and light colours and intensity for different experiments. As a result, all variables were adjusted while other factors remained constant in each experiment.

### Plant cultivation and growing conditions

**Figure 1:**
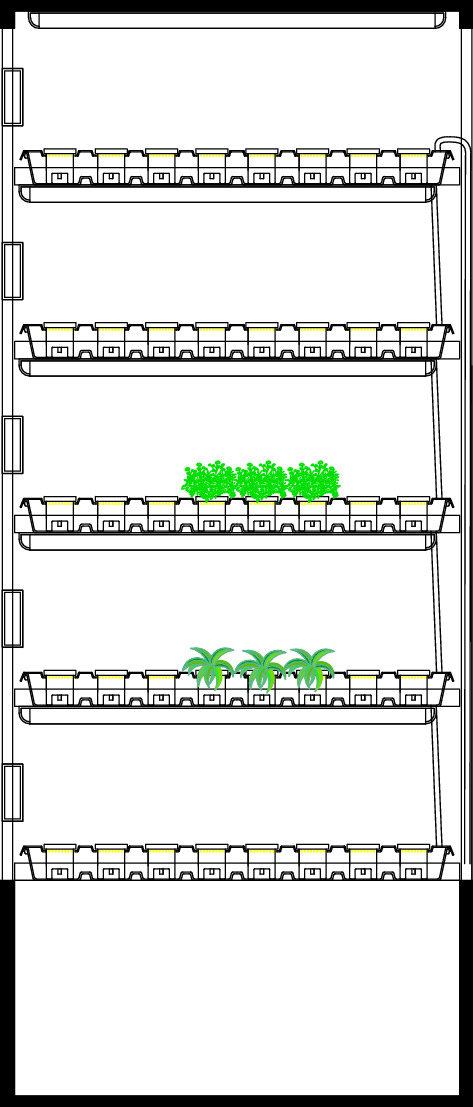
Placement of basil and lettuce in the vertical farms.

The seeds of Lettuce (*Lactuca sativa* L.) cultivar “Batavia- Caipira” (Enza Zaden, the Netherlands) first were placed and grown in a rockwool plug. The seeds of basil (*Ocimumbasilicum* L.) cultivar ‘’Emily’’(Enza Zaden, the Netherlands) have been sowed in plantation pots (9 cm H × 9 cm W × 9 cm L) filled with vermiculites. In germination phase pots were spraying with distilled water once a day and placed in dark closed heated boxes. The boxes of both lettuce and basil plants have been kept on a room with temperature of 24 ± 2 °C, 50–60% relative humidity and 450 ppm CO_2_. Finally, the germinated lettuce seeds were first transferred to rockwool blocks and placed on the shelves of the vertical farm. The germination seeds of basil, on the other hand, were directly transferred into the vertical farm. To ensure similar conditions in all experiments, as displayed in the schematic picture in Fig. 1, three pots of lettuce and basil were placed on the second and third shelves in the middle (Fig. 1).

The used nutrient solution in all experiments was prepared according to Hoagland and Arnon^43^, with the following formula: 136 g KH_2_PO_4_, 101 g KNO_3_, 236.15 g Ca(NO_3_)_2_·4H_2_O, 246.48 g MgSO_4_·7H_2_O, 2.86 g H_3_BO_3_, 1.86 g MnCl_2_·4H_2_O, 0.22 g ZnSO_4_·5H_2_O, 0.08 g CuSO_4_·5H_2_O, 0.02 g H_2_MoO_4_·H_2_O and 10 g Fe EDDHA per 1 litter tap water, with pH 6.5.

### Experimental design using Taguchi approach

**Figure 2:**
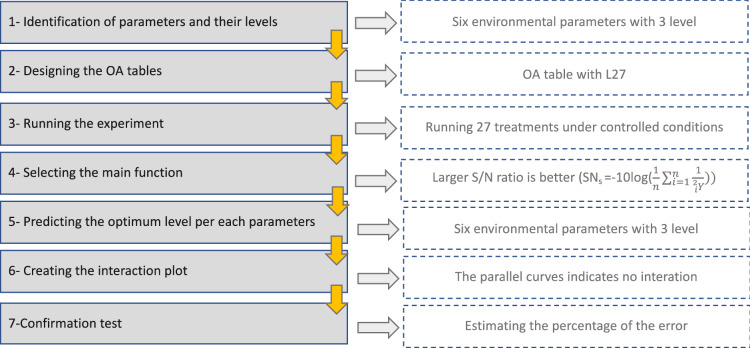
The used workflow to analyse the data using Taguchi approach in the current stud (S: Signal N: Noise ratio).

In this study we used Taguchi approach to select the combinations of environmental factors that needed to be experimented at the same time. With this aim, a workflow has been used which is shown in Fig. 2. The experimental design was performed using Minitab software (Minitab, LLC. version 16, 2021).

#### Step 1: identification of parameters and levels

As shown in Table 1, six factors that influence the growth parameters of plants grown in vertical farms were selected, including: relative humidity, LED type, day temperature, night temperature, CO_2_ concentration and EC of nutrition solution. The optimal levels of each factor depends on the cultivation systems used in the vertical farm and the cultivated plants. Therefore, three levels of each factor were selected based on a combination of previously published studies summarized in Table 2, as well as recorded data and observations resulting from preliminary analyses conducted on the vertical farms. In a previous study conducted by Hossein et al.^8^, it was found that an ebb-flow hydroponic system requires less electrical conductivity (EC) compared to other systems due to the floating of roots in the nutrient solution. This study helped us to determine the appropriate EC levels of 0.7, 0.9, and 1.2 for our experiment. Moreover, various studies have shown that the use of white LEDs supplemented with red and far-red lights can enhance plant growth parameters. Thus, to consider the effect of photosynthetic photon flux density (PPFD), we selected three levels of LED with combinations of white, red, and far-red, as well as only white light, and two PPFD levels. However, we could not find any suitable studies on the effects of humidity, temperature and CO_2_ levels in an ebb-flow system for basil and lettuce. Therefore, we selected three levels based on preliminary studies conducted before this experiment and recorded data available in Vegger B.V.

**Table 2:** The selected factors and levels for designing an experiment using Taguchi method in the present study.

Factors	Level 1	Level 2	Level 3
CO2 concentration (ppm)	400	600	800
LED type*	W122	WRF122	WRF244
EC of nutrient solution	0.7	0.9	1.2
Day temperature (°C)	15	22	26
Night temperature (°C)	10	15	20
Relative humidity	35%	55%	75%

*W white, *R* deep red, *F* far red, 122 and 244 indicate the amount of PPFM.

#### Step 2: designing the OA table

Accordingly, 27 experiments for 6 factors and 3 levels have been selected using Taguchi method which are summarized in Table 3. Each treatment with three replicates has been performed under controlled condition in a modular vertical farm unit.

**Table 3:** The selected trials designed by Taguchi method in the present studies.

Treatment	CO_2_	LED	EC	Day temp	Night temp	Humidity
1	1	1	1	1	1	1
2	1	1	1	1	2	2
3	1	1	1	1	3	3
4	1	2	2	2	1	1
5	1	2	2	2	2	2
6	1	2	2	2	3	3
7	1	3	3	3	1	1
8	1	3	3	3	2	2
9	1	3	3	3	3	3
10	2	1	2	3	1	2
11	2	1	2	3	2	3
12	2	1	2	3	3	1
13	2	2	3	1	1	2
14	2	2	3	1	2	3
15	2	2	3	1	3	1
16	2	3	1	2	1	2
17	2	3	1	2	2	3
18	2	3	1	2	3	1
19	3	1	3	2	1	3
20	3	1	3	2	2	1
21	3	1	3	2	3	2
22	3	2	1	3	1	3
23	3	2	1	3	2	1
24	3	2	1	3	3	2
25	3	3	2	1	1	3
26	3	3	2	1	2	1
27	3	3	2	1	3	2

#### Step 3: running the experiment

The 27 selected experiments (Table 3) have been conducted under controlled conditions (see Sections “Vertical farms and plant growth chambers” and “Plant cultivation and growing conditions”) in vertical farms. Lettuce and basil plants have been harvested 45 and 40 days after the transferring to the hydroponic system respectively. To collect the dried material, plants have been placed in the oven with 72 °C for 72 h^44^.

The growth parameters including number and area of leaves, the biomass weight of root and leaves (fresh/dry), and concentration of micro and macro nutrients [Nitrogen (N), Potassium (K), Calcium (Ca), Magnesium (Mg), Phosphorus (P), Iron (Fe), Zinc (Zu), Manganese (Mn) and Copper (Cu), Boron (B)]were measured in lettuce and basil dried materials.

The total chlorophyll of the third fully grown leaf were measured using the method described by Wellburn^45^. The 100 mg fresh leaf has been extracted using 5 ml acetone (80%), the solution Centrifuge for 10 min at 3800 rpm. The absorption of the solution has been measured by a spectrometer (Rayleigh VIS-7220G) at 663, 645. The Chlorophyll has been determined based on the following equations described by Wellburn^45^:

$${\text{Chla}} = \left[ {{12}.{25}\left( {{\text{A663}}.{2}} \right) - \left( {{\text{A646}}.{8}} \right)} \right] \times {\text{V}}/{1}000 \times {\text{W}}$$

$${\text{Chlb}} = \left[ {{25}.{51}\left( {{\text{A646}}.{8}} \right) - \left( {{\text{A663}}.{2}} \right)} \right] \times {\text{V}}/{1}000 \times {\text{W}}$$

$${\text{Chl}} = {\text{chlb}} - {\text{chla}}$$

The percentage of nitrogen concentration of each sample were measured by modified Kjeldahl method^44^. The results have been measured as % dry matter and mg kg^−1^ DW. The micro and macro elements of dried leaves were measured using ICP-OES (Thermo Fisher Scientific iCAP 6300) in the materials extracted by ash method^44^.

#### Step 4: selecting the main function

In all experiments, the signal-to-noise ratio (S/N) has been calculated to assess the quality of the response. In the Taguchi method, the signal refers to the desired response, while noise refers to the variation or random factors that can affect the response. Controllable factors are associated with the signal, while uncontrollable factors are associated with noise. To eliminate the effects of noise, it is important to conduct experiments in a controlled environment and to minimize the impact of external factors on the response. The method of controlling the conditions has been explained in Sections “Vertical farms and plant growth chambers” and “Plant cultivation and growing conditions” and includes techniques such as conducting experiments in a stable environment, using high-quality instruments, controlling environmental conditions to minimize the effects of external factors that are difficult to control. By minimizing the effect of noise, the Taguchi method can provide more accurate and reliable results, leading to improved performance and quality of the system being studied. Three different functions can be used for Taguchi analysis: (i) larger S/N ratio is better, (ii) smaller S/N ratio is better and (iii) nominal S/N ratio is better. Since in our study, the objective is maximizing the response, the first function (function i) was selected for further analysis.

#### Step 5: prediction the optimum level

The calculation, ranking the factors and finding the best conditions has been performed for each parameters including number and area of leaves, the biomass weight of root and leaves (fresh/dry), chlorophyll contents and concentration of micro and macro nutrients [Nitrogen (N), Potassium (K), Calcium (Ca), Magnesium (Mg), Phosphorus (P), Iron (Fe), Zinc (Zu), Manganese (Mn) and Copper (Cu), Boron (B)], has been determined using Minitab (Minitab, LLC. version 16, 2021). Mini tab ranked the studied factors based on calculated “delta” which is the difference between highest and lowest average response value of each parameter at different levels. First rank goes to the highest and last rank goes to the lowest delta.

#### Step 6: interaction plot

To study the interrelation between each pair of conditions using Taguchi approach, interaction plots were generated. The Minitab plots the level of one factor in x-axis and the level of another factor on y-axis, the factors are displaying with two lines in blue and red colour. No interaction between the parameters is observed when the created lines are almost parallel to each other.

#### Step 7: confirmation test

To validate the results, an experiment with the combination of optimum level per each condition predicted were conducted by the program with three replicates. According to the results we calculated the error rate of the experiment.

### Statement of compliance

The authors confirm that the experimental research on basil and lettuce plants, including the collection of samples that were performed in this study comply with relevant institutional, national, and international guidelines and legislation.

## Results

### Analysing the effect of environmental factors on lettuce and basil growth parameters

The growth parameters including number of leaves, leaf area, weight of fresh and dry leaf and root in all selected treatments have been measured (Table 5). The S/N ratio of different levels in basil and lettuce have been plotted in Appendix, Fig. A1. The ranking of calculated factors is summarized in Table 4.

**Table 4:**
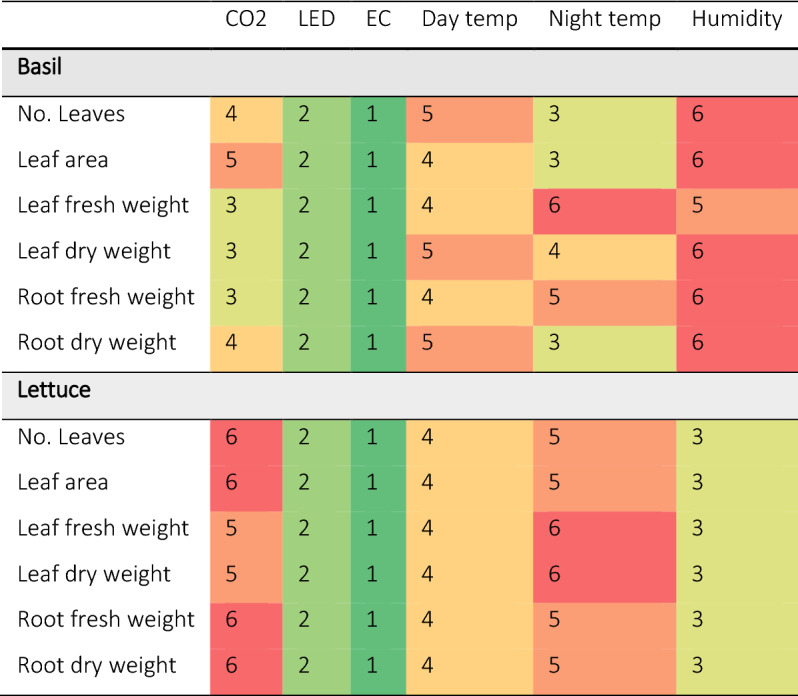
Ranking the effect of environmental factors on basil and lettuce growth parameters based on S/N ratios.

As it is shown in the Table 4, EC followed by LED light recipes ranked first among all factors for producing the biomass in lettuce. CO_2_ has the least impact on all measured growth parameters of lettuce except for dried and fresh weight of leaves. However, the night temperature has the lowest impact on the weight of fresh and dried leaves. The importance of humidity on all lettuce growth parameters is more than day/night temperature. Day temperature was more important than night temperature for measured growth parameters of lettuce.

EC and LED have the highest impact on all studied growth parameters of basil. Humidity has the lowest impact on all measured basil growth parameters except for leaf fresh weight. The ranks of day/night temperature and CO_2_ effectiveness on basil growth parameters varied from 3 to 5.

#### Analysing the effect of parameters on chlorophyll of lettuce and basil 

The effect of studied factors on the chlorophyll content in the selected conditions in lettuce and basil are indicated in Table 5 and Fig. A2 (see the Appendix). Based on the results, EC followed by LED are the most effective parameters on chlorophyll content. Humidity followed by CO_2_ and CO_2_ followed by Night temperature are the least effective parameters in basil and lettuce, respectively.

**Table 5:** The mean of calculated basil and lettuce growth parameters and chlorophyll in three replicates per each experiment.

Run	Plant	No. leaf	Leaf area	Fresh leave	Dry leave	Fresh root	Dry root	Chlorophyll
1	Lettuce	11	688	17.15	0.48	1.82	0.321	1.32
	Basil	4	20	1.82	0.02	0.44	0.018	0.94
2	Lettuce	14	872	21.72	0.67	2.14	0.367	1.68
	Basil	7	32	3.22	0.035	0.77	0.0315	1.64
3	Lettuce	17	1071	26.35	0.77	1.77	0.311	1.38
	Basil	8	34	3.16	0.04	0.88	0.036	1.89
4	Lettuce	16	1001	24.85	0.72	2.46	0.42	1.95
	Basil	5	21	2.47	0.025	0.65	0.0225	1.18
5	Lettuce	18	1120	27.77	0.81	3.1	0.455	2.26
	Basil	11	45	5.21	0.055	1.21	0.0495	2.63
6	Lettuce	19	1188	29.45	0.86	2.67	0.421	2
	Basil	11	48	4.62	0.055	1.34	0.0495	2.55
7	Lettuce	18	1127	28.10	0.82	2.82	0.472	2.22
	Basil	17	72	7.82	0.085	1.87	0.0765	3.91
8	Lettuce	17	1063	26.35	0.77	2.88	0.431	2.04
	Basil	18	76	8.1	0.09	2.11	0.081	4.28
9	Lettuce	19	1188	29.45	0.86	2.62	0.432	2.11
	Basil	18	78	7.52	0.09	2.08	0.081	4.32
10	Lettuce	22	1377	34.10	1.01	4.32	0.682	3.37
	Basil	12	51	5.62	0.06	1.32	0.054	2.85
11	Lettuce	20	1245	30.91	0.90	2.51	0.405	2.1
	Basil	9	37	4.15	0.045	1.13	0.0405	2.12
12	Lettuce	17	998	26.35	0.77	2.72	0.421	2.12
	Basil	12	49	5.44	0.06	1.32	0.054	3.12
13	Lettuce	16	992	24.62	0.75	2.58	0.385	1.97
	Basil	15	62	6.78	0.075	1.67	0.0675	3.27
14	Lettuce	18	1122	27.90	0.82	2.54	0.325	1.68
	Basil	14	57	6.45	0.07	1.44	0.063	3.34
15	Lettuce	14	870	22.00	0.63	2.24	0.35	1.71
	Basil	15	63	6.92	0.075	1.57	0.0675	3.61
16	Lettuce	19	1172	29.50	0.86	3.15	0.476	2.32
	Basil	9	38	4.5	0.045	1.27	0.0405	2.17
17	Lettuce	17	1063	26.37	0.78	2.44	0.362	1.75
	Basil	7	31	3.11	0.035	0.82	0.0315	1.67
18	Lettuce	13	820	20.15	0.59	2.18	0.336	1.61
	Basil	12	53	5.35	0.06	1.62	0.054	2.77
19	Lettuce	15	941	23.51	0.69	1.92	0.275	1.33
	Basil	13	55	5.62	0.067	1.72	0.0585	3.12
20	Lettuce	11	688	17.05	0.50	1.86	0.278	1.32
	Basil	11	47	4.68	0.057	1.08	0.0495	2.56
21	Lettuce	14	875	21.67	0.63	2.34	0.352	1.7
	Basil	16	67	7.21	0.08	1.92	0.072	3.77
22	Lettuce	11	685	17.20	0.49	1.96	0.302	1.33
	Basil	11	48	5.21	0.055	1.44	0.0495	2.47
23	Lettuce	10	625	15.42	0.45	1.67	0.255	1.2
	Basil	9	39	4.05	0.044	1.34	0.0405	2.32
24	Lettuce	12	751	18.60	0.55	2	0.32	1.46
	Basil	12	52	5.32	0.06	1.56	0.054	2.91
25	Lettuce	35	2172	52.50	1.57	5.31	0.882	4.25
	Basil	13	55	5.8	0.064	1.7	0.0585	3.12
26	Lettuce	24	1482	37.20	1.08	4.12	0.62	2.88
	Basil	13	56	6.1	0.065	1.87	0.0585	2.97
27	Lettuce	28	1750	43.40	1.26	4.62	0.7	3.26
	Basil	14	61	6.23	0.07	1.54	0.063	3.34

#### Analysing the effect of parameters on micro- and macro-elements of lettuce and basil

The effect of studied factors on the micro and macro elements are illustrated in Table 6 and Figs. A3 and A4 (see the Appendix).

**Table 6:** The mean of measured Micro and elements in three replicates per each experiment.

Run	Plant	Fe	Zn	Cu	Mn	N	P	K	Ca	Mg	B
1	Lettuce	47.80	10.20	3.59	25.07	1.21	0.13	1.51	0.60	0.30	15.37
	Basil	66.50	18.30	5.19	27.87	1.30	0.15	1.20	0.68	0.39	19.76
2	Lettuce	63.13	12.78	4.50	33.11	1.60	0.17	1.89	0.76	0.38	20.31
	Basil	62.81	17.59	4.99	31.67	1.25	0.17	1.16	1.18	0.37	22.40
3	Lettuce	47.34	10.84	3.81	24.83	1.20	0.13	1.61	0.84	0.32	15.23
	Basil	96.06	20.27	5.75	27.02	1.44	0.17	1.33	1.36	0.43	19.0
4	Lettuce	64.33	12.01	4.23	33.74	1.63	0.18	1.78	0.71	0.36	20.69
	Basil	90.52	15.76	4.47	23.43	1.12	0.15	1.04	0.85	0.33	16.54
5	Lettuce	73.95	13.80	4.86	38.78	1.87	0.20	2.04	0.82	0.41	23.79
	Basil	140.58	25.61	7.27	43.07	1.82	0.25	1.69	1.90	0.54	30.27
6	Lettuce	65.83	12.29	4.32	34.53	1.67	0.18	1.82	0.93	0.36	21.18
	Basil	136.30	20.77	5.89	30.40	1.48	0.20	1.37	1.84	0.44	21.50
7	Lettuce	119.50	27.11	9.54	62.67	3.53	0.38	4.02	1.32	0.80	38.44
	Basil	209.00	31.85	9.04	42.46	2.26	0.30	2.10	2.82	0.67	30.10
8	Lettuce	127.45	25.72	9.05	66.85	3.23	0.35	3.81	1.52	0.76	41.0
	Basil	242.00	38.70	10.98	57.00	2.75	0.37	2.55	3.09	0.81	40.12
9	Lettuce	117.20	24.92	8.77	61.47	2.77	0.30	3.69	1.58	0.74	37.70
	Basil	230.92	35.19	9.99	51.93	2.50	0.34	2.31	3.11	0.74	36.54
10	Lettuce	55.70	20.20	7.11	29.21	3.02	0.33	2.99	1.20	0.60	17.92
	Basil	121.92	22.80	6.47	35.47	1.62	0.22	1.50	2.05	0.48	24.87
11	Lettuce	37.60	13.35	4.70	19.72	1.81	0.20	1.98	0.89	0.40	12.10
	Basil	113.32	17.27	4.90	23.02	1.23	0.17	1.14	1.53	0.36	16.10
12	Lettuce	42.29	13.49	4.75	22.18	1.83	0.20	2.00	0.80	0.40	13.60
	Basil	194.89	25.61	7.27	34.15	1.82	0.25	1.69	2.25	0.54	2a3.80
13	Lettuce	143.09	26.71	9.40	75.05	3.62	0.39	3.96	1.38	0.79	46.03
	Basil	177.34	22.80	6.47	31.67	1.62	0.22	1.37	2.36	0.48	22.30
14	Lettuce	74.85	13.97	4.92	39.26	1.90	0.20	2.07	1.28	0.41	24.08
	Basil	178.53	24.35	6.91	35.47	1.73	0.21	1.47	2.41	0.51	24.90
15	Lettuce	78.16	14.59	5.13	40.99	1.98	0.21	2.16	0.86	0.43	25.14
	Basil	192.96	26.04	7.39	40.53	1.85	0.25	1.59	2.60	0.55	28.35
16	Lettuce	81.76	15.26	5.37	42.88	2.07	0.22	2.26	1.10	0.45	26.30
	Basil	115.99	17.67	5.02	23.57	1.26	0.17	1.16	1.56	0.37	16.50
17	Lettuce	61.62	11.50	4.05	32.32	1.56	0.17	1.70	1.68	0.34	19.82
	Basil	101.60	15.48	4.39	20.64	1.10	0.15	1.02	1.20	0.33	14.50
18	Lettuce	55.91	10.44	3.67	29.32	1.42	0.15	1.55	0.62	0.31	17.99
	Basil	148.06	22.56	6.40	30.08	1.60	0.22	1.48	2.00	0.47	21.12
19	Lettuce	79.21	14.79	5.20	41.54	2.01	0.22	2.19	1.88	0.44	25.48
	Basil	166.77	25.41	7.21	33.88	1.81	0.24	1.67	2.25	0.53	13.80
20	Lettuce	78.46	14.65	5.15	41.15	1.99	0.21	2.17	0.87	0.43	25.24
	Basil	136.84	20.85	5.92	27.80	1.48	0.20	1.37	1.85	0.44	19.50
21	Lettuce	88.98	16.61	5.84	46.67	2.25	0.24	2.46	0.98	0.49	28.62
	Basil	201.52	16.47	4.67	21.96	1.17	0.16	1.08	2.72	0.35	15.40
22	Lettuce	83.50	6.12	2.15	43.79	0.83	0.09	0.91	0.36	0.18	26.86
	Basil	132.03	20.12	5.71	26.82	1.43	0.19	1.32	1.78	0.42	15.87
23	Lettuce	57.24	7.07	2.49	30.02	0.96	0.10	1.05	0.42	0.21	18.41
	Basil	124.01	18.90	5.36	25.19	1.34	0.18	1.24	1.67	0.40	17.70
24	Lettuce	75.50	6.62	2.33	39.60	0.90	0.10	0.98	0.39	0.20	24.29
	Basil	155.55	22.24	6.31	29.65	1.58	0.21	1.46	2.10	0.47	20.82
25	Lettuce	143.70	24.69	8.69	75.37	3.35	0.36	3.66	1.64	0.73	46.23
	Basil	158.87	21.39	6.07	28.52	1.52	0.20	1.34	2.25	0.45	20.10
26	Lettuce	121.30	18.85	6.63	63.62	2.56	0.28	2.79	1.12	0.56	39.02
	Basil	158.75	18.44	5.23	24.58	1.31	0.18	1.15	2.14	0.39	17.30
27	Lettuce	117.84	22.00	7.74	61.80	2.99	0.32	3.26	1.30	0.65	37.91
	Basil	178.53	23.50	6.67	31.34	1.67	0.22	1.48	2.41	0.49	22.0

The ranking of environmental factor effects on macro and micro elements are variable (Table 7). However, EC can be considered the most effective factors for majority of elements, except for Fe and Mn of lettuce.

**Table 7:**
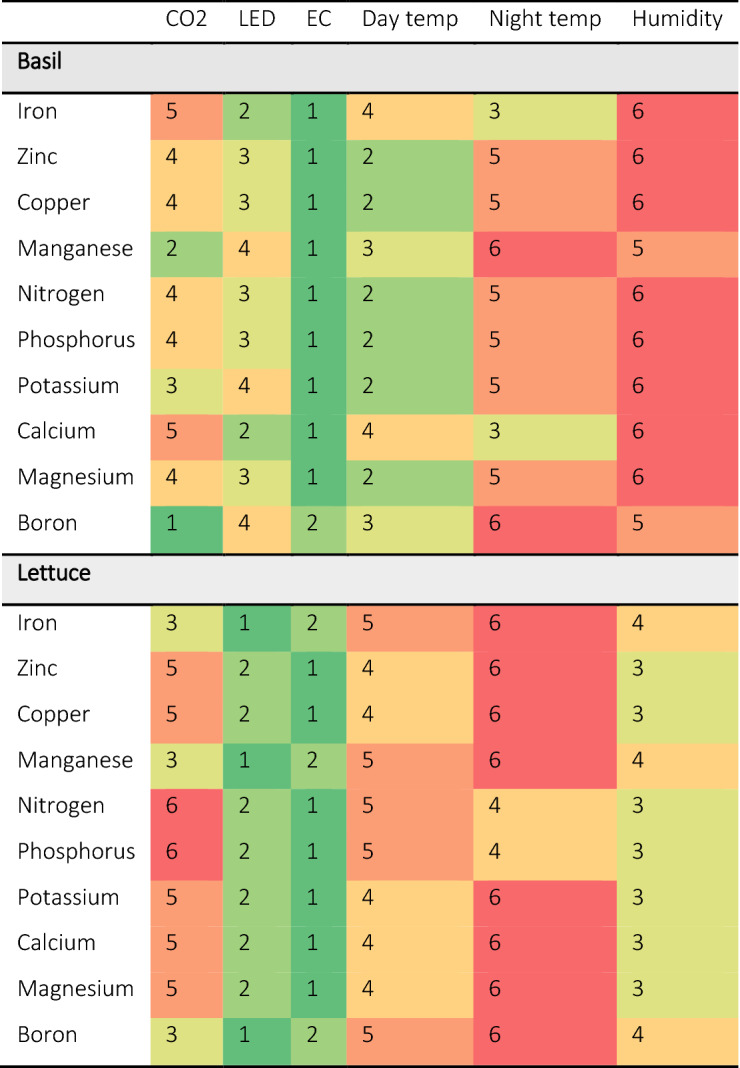
Ranking the effect of environmental factors on micro and macro elements of basil and lettuce plants based on S/N ratios.

### Predicting the best factor levels for lettuce and basil production 

The best level of studied environmental factors on growth parameters, chlorophyll content as well as micro/macro elements in the leaves of the basil and lettuce plants have been summarized in Table 8.

**Table 8:** Predicted and expected value obtained from Taguchi approach and confirmation test.

Parameters	S/N	Expected value	Predicted value	Error (%)
Basil				
No. leaves	28.30	21.04	20.78	1.25
Leave area	40.85	90.99	89.11	2.07
Fresh leaves	21.09	9.15	9.04	1.17
Dry leaves	17.65	0.11	0.10	2.68
Fresh roots	9.78	2.53	2.45	3.0
Dry roots	18.63	0.10	0.09	3.18
Chlorophyll	15.96	5.13	4.98	2.84
Nitrogen	9.31	2.81	2.74	2.5
Iron	50.10	269.85	266.07	1.4
Zinc	32.28	39.28	38.60	1.75
Copper	21.34	11.24	10.95	2.55
Manganese	35.87	58.84	57.10	2.96
Phosphorus	7.97	0.38	0.37	3.23
Potassium	8.63	2.56	2.64	1.07
Calcium	13.12	5.63	3.59	0.98
Magnesium	1.26	0.82	0.81	0.88
Boron	32.98	40.98	40.52	1.11
Lettuce				
No. leaves	31.24	34.10	33.33	2.26
Leave area	67.01	2117.41	2059.39	2.74
Fresh leaves	34.84	51.08	50.45	1.24
Dry leaves	4.29	1.51	1.50	0.82
Fresh roots	15.54	5.34	5.28	1.08
Dry roots	0.39	0.86	0.84	2.35
Chlorophyll	13.74	4.24	4.17	1.86
Nitrogen	14.77	4.36	4.27	2.03
Iron	47.21	182.91	177.43	3.0
Zinc	32.51	33.36	32.97	1.17
Copper	23.44	11.72	11.60	1.02
Manganese	41.60	93.97	93.06	0.97
Phosphorus	4.56	0.47	0.46	1.82
Potassium	15.93	4.94	4.88	1.10
Calcium	8.02	2.0	1.95	2.52
Magnesium	1.95	1.0	0.98	2.11
Boron	37.36	57.97	57.07	1.37

For lettuce, the optimum LED recipe which has the best effect on measured parameters in all experiments was ‘level 3’ which is the combination of white with extra far red and deep red and 244 PPFM. The best EC for growth parameters and chlorophyll of lettuce was 0.9 and for its micro and macro elements were 1.2. The best estimated day/night temperature affecting the measured parameters of lettuce was 15/10. Best CO_2_ level for lettuce growth parameters, N, P, Ca, Chl is 600; for Zn, Cu, K, Mg is 400 and for Fe, B and Mn is 800. The relative humidity of 75% estimated to be the best for increase the amount of Ca in the leaves, Number, area, and fresh weight of leaves of lettuce plants. For other parameters the best relative humidity was 55%.

Based on the results, the best EC, LED, and relative humidity for all parameters of basil are 1.2, WRF244 and 55%. The temperature of 26/20 is the best combination of day/night temperature for measured basil parameters. CO_2_ of 400 ppm predicted to be the best level for basil growth parameters and chlorophyll content. The best CO_2_ level for increasing the macro and micro elements in basil are 800 ppm, except for Ca and Fe.

### Interrelations between environmental factors

The synergistic effect of different levels of environmental factors per growth parameters, chlorophyll content and measured elements has been studied by generating the interaction plots. Based on the created plots (see an example in Appendix, Fig. A5), among almost all factors there was interrelations (EC and light, light and relative humidity, EC and temperature, temperature and relative humidity, CO_2_ and relative humidity, CO_2_ and EC as well as CO_2_ and light). However, there was no synergistic effect between EC and relative humidity on measured growth parameters of lettuce and basil.

### Confirmation test

**Figure 3:**
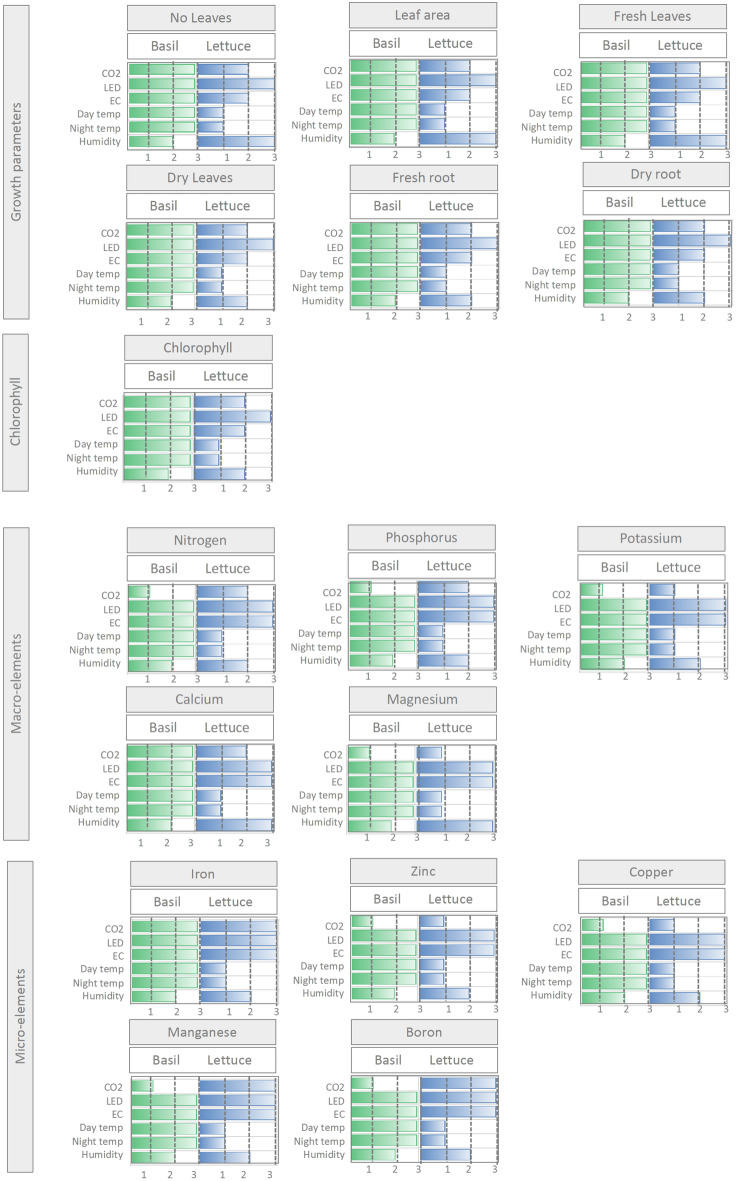
Predicted best factor per each parameter.

To validate the result of Taguchi test, we run the confirmation tests with predicted optimum level of environmental factors for each plant parameter (see Fig. 3). The predicted plant parameters using Taguchi test and the result of confirmation test have been summarized in Table 8. We also calculated the error ratio between predicted and real number obtained from confirmation test (expected value – predicted value/expected value). The calculated error rate between expected and predicted value for all plant parameters in basil and lettuce are 0.88–3.23 and 0.82–3.0 respectively.

## Discussion

In this study, the Taguchi method was employed to determine the optimal environmental conditions for cultivating lettuce and basil plants, as traditional experiments face challenges when concurrently examining the influence of multiple factors. While the Taguchi method is a powerful tool for optimization, other experimental design methods, such as Technique for Order Preference by Similarity to Ideal Solution (TOPSIS) and Particle Image Velocimetry (PIV), also offer valuable insights in optimization and decision-making processes. These methods rely on statistical and mathematical principles to analyze data and predict outcomes^46^, demonstrating their applicability across a range of scenarios. However, the Taguchi method presents several advantages for experimental studies like the one presented in this paper, making it a particularly well-suited approach for exploring the multifaceted relationships between environmental factors and plant growth in vertical farming systems. he Taguchi method offers several notable advantages when applied to experimental studies, such as the one presented in this paper. First and foremost, this method allows for the efficient examination of multiple factors simultaneously, reducing the number of experiments required, which in turn saves both time and resources. Furthermore, the Taguchi method provides robust results even with a smaller sample size, ensuring a high level of accuracy and reliability. Additionally, its capacity to effectively identify interactions between factors and quantify their individual and combined effects on the desired outcome makes it particularly valuable for studies involving complex systems, such as vertical farming environments.

In the present study, to make the study practical and cost-effective, we utilized the Taguchi method, which allowed us to minimize the number of experiments needed to identify the optimal combination of factors affecting plant growth parameters. By employing this method, we were able to run only 18 combinations of factors, saving time, labour, and resources^15,47^. However, it is important to note that the results obtained from Taguchi optimization are limited to the specific levels and combinations of factors tested during the optimization process, and careful interpretation of the results is necessary^15,47^. However, to overcome this limitation, we used the Minitab program to calculate the sample size and design a larger OA table compared to other programs, allowing us to run 27 experiments instead of 18.

Furthermore, the Taguchi method is designed to be robust to small variations in experimental conditions, which can minimize the effects of noise and other random factors that can affect the results of the experiment. This improved the reliability of the results and reduced the risk of false conclusions due to random fluctuations^15,47^. Additionally, conducting a confirmation test and calculating the error rate increased the robustness of the analysis.

While, Taguchi method has been used in different fields such as medicine^48^ and engineering^16,49,50^, its application in agriculture is relatively novel and limited to a few numbers of conducted research. By utilizing this method, we were able to identify the most critical factors affecting plant growth parameters by analysing the variation in response variables caused by individual factors and their interactions. This provided insights into which factors were essential for optimizing plant growth and which factors could adjusted with less impact on the response variables^51,52^. This study is a novel study in the field of controlled-environment agriculture as it explores the influence of six different factors including CO_2_, day/night temperature, EC, LED type and relative humidity with three different levels at same time. The following sections discuss the findings related to each studied environmental factor separately and the last section explains the interrelation of different environmental factors with each other.

### CO_2_ level

The response of plants to CO_2_ depends on the plant species and other environmental factors such as temperature, light, nutrients and humidity and heat stress^35,53–56^. According to the results of this study, CO_2_ has less impact on the lettuce growth parameters and the chlorophyll content of lettuce in compared to the other factors. The impact of CO_2_ on growth parameters of Basil and micro/macro elements of both plants was variable. The analyses showed that while the effect of CO_2_ on growth parameters of basil ranked mostly 3 or 5, this effect on micro and macro elements of both plants ranked 1–6. The low impact of CO_2_ on plant measured parameters in compared to other environmental factors such as nutrients and temperature has been observed previous studies as well^41,57^. One reason for least impact of CO2 in lettuce is that lettuce is a cool-season crop, and its growth is primarily influenced by temperature and light. Therefore, while increased CO2 levels may enhance photosynthesis, the growth of lettuce may be limited by other factors, such as the availability of nutrients. This is also observed in other studies that were done on such crops^41^.

While the highest level of CO_2_ in this study (800 ppm) is the best predicted CO_2_ level for basil biomass and chlorophyll production, most of micro and macro elements except for Ca and Fe increased in the lowest CO_2_ level of this study (400 ppm). The predicted optimum level for lettuce growth parameters and macro elements (except for Mg) is 600 ppm and for micro element (except for Fe, B, Mn) and Mg is 400 ppm.

CO_2_ can consider as a “gas fertilizer”^35^ in greenhouses to promote the yield and biomass production and plant resistance against environmental stress by enhancing the photosynthesis process, soluble sugars, and antioxidant^9,35,36,58^. However, elevated CO_2_ is observed to cause reduction of some nutrient elements, proteins, chlorophyll content and nitrates. The reduction of nutrient elements can be due to dilution effect resulted from higher amount of biomass^35^. Furthermore, the movement of nitrogen or non-structural carbohydrates during the different plant organs and basil chlorosis symptom or lettuce tip burn symptoms due to elevated CO_2_ can led to reducing the chlorophyll content^9,34^. The increased risk of tip-burn of leaves by increasing the CO_2_ level was also observed in this study. However, this was not the focus of our study and further research on the relationship between the level of CO_2_ and tip burn can be beneficial for practitioners.

### Night and day temperature

The optimum range of studied temperature was different for lettuce and basil. It is already known that the temperature within the optimum range can increase the growth parameters by elongation and increasing the thickness of tissues due to promoting the plant metabolic process^59^. The temperature out of the optimum range can suppress the growth parameters, decline the yields, and creates chlorosis and discoloration of leaves^39,59^. In this study, the best predicted day/night temperature for lettuce and basil was 15/10 and 26/20 °C, respectively. This finding is in line with the previous studies which introduce basil and lettuce as the warm and cold climate plants^60^. The high temperature can accelerate the seed production and results in very tall lettuce. However, basil is a hot climate plant which is sensitive to low temperature. Low temperature cause very serious damage and specially in initial growth phase, heat is deadly for the basil plants^5,61^. According to our results the importance of day temperature for all lettuce growth parameters, some of basil growth parameters and most of nutrient elements of both plants are more than night temperature. Photosynthesis and respiration process which occurred during day and night, respectively, increases the temperature. The importance of day temperature compared to night temperature has been observed in previous studies^62^. During the day, when photosynthesis is most active, temperature has a significant impact on plant growth as it affects the rate of photosynthesis and nutrient uptake. High temperatures during the day can lead to increased transpiration rates and water stress, negatively impacting plant growth. Conversely, at night, plant metabolism slows down, and the temperature has a less significant impact on growth.

As such, finding the best temperature to keep the balance between photosynthesis and respiration process are very important. Our study indicates that that the difference between predicted optimum day and night temperature which is named “DIF”, is positive with higher day temperature than night temperature. The DIF, influence the plant growth process and plant’s structure. So, positive DIF with higher temperature in day and lower temperature during night lead to increasing of photosynthesis and reducing of respiration which can result in higher biomass production and nutrient concentration^63,64^. Additionally, lower temperature at night led to less energy consumption for keeping the greenhouse warm during night. However, negative DIF cause the reduction of stem elongation and thickness which is favourable for production of some plants such as ornaments, tomato, and potato. The reduction of elongation occurred due to reducing the hormones^64,65^.

### Relative humidity

Relative humidity (RH) or the amount of water in the air, is a factor that influences plant’s transpiration which has been studied in this paper. Circulating the water taken by root and evaporated via leaves can be affected by relative humidity. The optimum level of RH per each plant can vary depends on environmental temperature. The optimum level of RH for lettuce and basil was 75% and 55% respectively. The effect of relative humidity for lettuce was more than basil. Higher or lower relative humidity influence the plant physiological process specially photosynthesis and transpiration. The RH higher than optimum range, provides suitable conditions for distribution of common fungal and bacterial diseases and pests. In the lettuce plant high RH creates tip burn due to less transpiration and less calcium up taking. The low humidity causes more inspiration, less photosynthesis and dehydration resulted in wilting the plants and led to reducing the desirability of the products for the market^37,66^.

### LED lights

The light source is one of the last two factors which has the highest impact on most of measured plant parameters. According to our study and previous research, white LEDs which is combination of different light including red and blue, are very beneficial for plants when proper supplementary lights are added and optimal light intensity or photon flux density (PPFD) is adjusted^27,28,67^. In this study we used three types of LED, white without additional lights, white with deep red light (660 nm) and far-red light (700–800 nm) with 122 PPFD and the same lights but with 244 PPFD. Adding far red and deep red can promote growth parameters. Deep red enhances biomass weight and the far-red, which is out of light visible range, can boost the effect of other LED lights by increasing the concentration of elements produced during photosynthesis process^68,69^. However, the best predicted level of LED light recipe in this study is combination of white light with deep red and far red when the PPFD is doubled (PPFD = 244).

### Electrical conductivity

EC is a very important factor which need to be optimized before and during cultivation of any plant^8^. Although, the less amount of nutrient elements results in preventing of plant growth, extra dosage of nutrients can be toxic and is not economic^70,71^. Previous study^8^, found that EC of 1.2 and 0.9 dS m^−1^ as the optimum level for increasing the growth parameters of basil and lettuce, respectively. In agreement with that study, the best EC for growth parameter and chlorophyll content was predicted to be 1.2 and 0.9 for basil and lettuce. However, the optimum EC for all micro and macro elements of both plants was 1.2 dS m^−1^. Almost for all parameters (except for a few nutrient elements) EC was the most important factor affecting the lettuce and basil growth parameters.

### Interrelation of different environmental factors

According to generated interaction plots most factor affecting the plant parameters have synergistic effect with each other except for EC and relative humidity on measured growth parameters of lettuce and basil. Previous research proved interactions between environmental parameters affecting the lettuce and basil production in controlled environment such as interactions between light and temperature^42^, CO_2_ and temperature^41^ and EC and light^33^. Our result confirms existence of interactions between the above-mentioned factors and showed interrelations between other factors such as EC and light, light and relative humidity, EC and temperature, temperature and relative humidity, CO_2_ and relative humidity, CO_2_ and EC as well as CO_2_ and light.

## Conclusion

This study presents a novel approach to examining the simultaneous effects of six environmental factors in vertical farms equipped with ebb-flow hydroponic systems and analysing their interrelations using the Taguchi method. The results demonstrate the intricate interconnections among these factors, emphasizing the importance of considering their combined influence when optimizing growth parameters in controlled-environment agriculture. The low error rate (1.0 to 3.2) observed between the expected values derived from confirmation tests and the predicted values calculated through Taguchi analysis indicates the successful optimization of environmental factors using this method. These findings suggest that the Taguchi method holds promise as an effective tool for future research aimed at identifying optimal environmental conditions for various plant species and cultivation systems, ultimately enhancing the productivity and sustainability of controlled-environment agriculture.

## Supplementary Information

Supplementary Figures.

## Data Availability

The datasets generated during the current study are available from the corresponding author on reasonable request.
