# Supplementary material for: Optimizing growth conditions in vertical farming: enhancing lettuce and basil cultivation through the application of the Taguchi method
Source: Sci Rep. 2023 Apr 25;13:6717. doi: 10.1038/s41598-023-33855-z (PMC10130051; doi:10.1038/s41598-023-33855-z)
Supplement: Supplementary file 1 — Supplementary Figures. [file 41598_2023_33855_MOESM1_ESM.docx]

Supplementary Materials

**Figure A1.** The SN ratios of factors on a) the number of leaves; b) leave area; c) fresh weight of leaves; d) dry weight of leaves; e) fresh weight of root and f) dry weight of root in basil and lettuce plants. The numbers 1-6 are related to: CO2, LED, EC, Day temperature, Night temperature and humidity.

**Figure A2**. The SN ratios of factors on chlorophyll contents in a) basil and b) lettuce. The numbers 1-6 are related to: CO2, LED, EC, Day temperature, Night temperature and humidity.

**Figure A3.** The SN ratios of factors on macro-elements including a) Nitrogen (N); b) Phosphorus (P); c) Potassium (K); d) Calcium (Ca) and e) Magnesium (Mg) in basil and lettuce. The numbers 1-6 are related to: CO2, LED, EC, Day temperature, Night temperature and humidity.

**Figure A4.** The SN ratios of factors on micro-elements including a) Iron (Fe); b) Zinc (Zn); c) Copper (Cu); d) Manganese (Mn) and e) Boron (B). The numbers 1-6 are related to: CO2, LED, EC, Day temperature, Night temperature and humidity.

A)

 B)

**Figure A5.** A) An example of the interaction plots for the studied environmental factors and growth parameters – this example shows the interaction plot of effect of CO2 and LED on the number of lettuce leaves. B) interaction plot B with almost parallel lines confirms the lack of interaction between EC and humidity on fresh weight leaves of basil.
